# Anxiety and Depressive Symptoms Post-COVID-19 Pandemic Onset in Solid Organ Transplant Recipients: Canadian Repeated Cross-Sectional Study

**DOI:** 10.3390/jcm14144920

**Published:** 2025-07-11

**Authors:** Jad Fadlallah, Vishva Shah, Ana Samudio, Tom Blydt-Hansen, Istvan Mucsi

**Affiliations:** 1 Ajmera Transplant Center, Division of Nephrology, University Health Network, Toronto, ON M5G 2N2, Canada; jad.fadlallah@uhn.ca (J.F.); vishva.shah@mail.utoronto.ca (V.S.); ana.samudio@uhn.ca (A.S.); 2 Division of Nephrology, Department of Pediatrics, University of British Columbia, Vancouver, BC V6H 3V4, Canada; tom.blydthansen@cw.bc.ca

**Keywords:** COVID-19 pandemic, solid organ transplant recipients, anxiety, depression

## Abstract

**Background:** Solid Organ Transplant Recipients (SOTRs) face an elevated risk of Sars-CoV-2 infection and poor outcomes if they contract the infection. This can induce or exacerbate anxiety and depressive symptoms. We used the Patient-Reported Outcomes Measurement Information System (PROMIS) Anxiety (A) and Depression (D) scores to conduct a repeated cross-sectional (“pseudo-longitudinal”) comparison of SOTRs’ anxiety and depressive symptoms before and after the COVID-19 pandemic onset. **Methods:** This secondary analysis used cross-sectional data from a convenience sample of adult SOTRs (kidney, kidney–pancreas, and liver) recruited between 2016 and 2024. The exposure was categorized as follows: “Pandemic Experience” was categorized as PRE (pre-pandemic reference; transplanted and anxiety and depressive symptoms assessed pre-pandemic onset), POST-1 (transplanted before and assessed after onset), and POST-2 (transplanted and assessed after onset). The outcomes were PROMIS-A and PROMIS-D scores. The differences were assessed using multivariable linear regression-estimated means. **Results:** Of the 816 participants, 588 (72%) were PRE, 135 (17%) were POST-1, and 93 (11%) were POST-2. In the fully adjusted model, the POST-2 group had significantly higher PROMIS-A scores (more severe symptoms) compared with PRE (adjusted mean [95% CI]: 54.2 [52.3; 56.1] vs. 51.7 [50.9; 52.4], *p* = 0.02). The proportion of patients with potentially clinically significant anxiety was also higher in the POST-2 group, compared with PRE (OR [95%CI] 1.59 [1.0; 2.5]). The PROMIS-A scores were similar between PRE and POST-1, and between POST-1 and POST-2. The PROMIS-D scores were not different across the exposure groups. **Conclusions:** SOTRs transplanted after the pandemic onset experienced more anxiety but similar depression symptoms compared with pre-pandemic levels. Future research should explore mental health support for SOTRs during crisis situations involving infectious risk.

## 1. Introduction

Solid Organ Transplant Recipients (SOTRs) are at a higher risk of infections and subsequent poor outcomes compared with the general population [1,2]. This vulnerability was highlighted at the onset of the COVID-19 pandemic. Studies have reported a higher incidence of Sars-CoV-2 infections [3]: 75% of SOTRs diagnosed with COVID-19 disease required hospital admission, of which 40% required intensive care and 30% required mechanical ventilation [4,5,6]. Additionally, SOTRs experienced an overall higher rate of COVID-19-related mortality compared with the general population [7]. The disproportionate impact of COVID-19 among SOTRs can be attributed to their immunocompromised condition, presence of comorbidities, and reduced vaccine effectiveness [8,9].

The prevalence of anxiety or depression in the first year following kidney or liver transplantation averages around 20–30% [10,11,12], and can reach up to 60% over the first several years [13]. This is often related to the chronic stress of managing their condition, the side effects of immunosuppressive medications, and the psychological burden of dependency on a transplant for survival [14,15].

Following the COVID-19 pandemic onset, the risk of contracting the SARS-CoV-2 virus has become an additional source of anxiety for SOTRs [16,17]. In addition, patients at all stages of their transplant journey experienced delays to their pre-transplant assessments and in accessing their healthcare team, which also had negative effects on mental well-being [18]. Furthermore, transplant recipients faced psychosocial stressors also affecting the general population during COVID-19, which included social distancing, masking, and lockdowns; economic disruptions such as unemployment and financial downturns; political unrest; increased substance use with related health consequences; and interruptions in peer support meetings and mental health treatment programs [19]. Anxiety and depressive symptoms are associated with a poor quality of life, increased mortality, and higher rates of graft rejection among SOTRs [11,13].

Anxiety and depressive symptoms can be assessed through patient-reported outcome measures (PROMs), which are standardized questionnaires that translate the patient voice into quantitative scores [20]. The Patient-Reported Outcomes Measurement Information System (PROMIS) tool offers a precise and efficient method of measuring anxiety and depression symptoms, providing clinically actionable scores [21,22,23,24].

To our knowledge, only a few studies have quantitatively analyzed anxiety and depressive symptoms among SOTRs in relation to the COVID-19 pandemic. Furthermore, these studies were not conducted with a reference pre-pandemic cohort nor with Canadian SOTRs. In this study, we use PROMIS Anxiety (PROMIS-A) and Depression (PROMIS-D) item bank scores to conduct a repeated cross-sectional comparison of anxiety and depressive symptoms among SOTRs before and after the onset of the COVID-19 pandemic.

## 2. Materials and Methods

### 2.1. Study Design, Participants, and Data Sources

This secondary analysis uses a repeated cross-sectional design, also referred to as a “pseudo-longitudinal” approach [25,26], in which data is collected from independent cross-sectional samples at distinct time periods to examine the differences in population characteristics across time. The analysis is based on cross-sectional data from the Comprehensive Psychosocial Research Data System (Co-PreDS), a research database established by the Kidney Health Education and Research Group (KHERG) in Toronto, Ontario, and approved by the University Health Network (UHN) Research Ethics Board (REB #17-5916 on 14 February 2018). The data included in this secondary analysis originates from primary studies validating the PROMIS tools among prevalent (transplant > 30 days before enrollment) SOTRs, with participants recruited between April 2016 and April 2024 from post-transplant outpatient clinics of the Ajmera Transplant Centre at the UHN in Toronto, Ontario, Canada. The primary studies were approved by the UHN research ethics boards (REBs) (#15-9645 on 25 October 2023 and #19-5097 on 9 July 2019) and by the REBs of the participating hospitals (#17-0061 on 22 December 2017, #2016-003-M on 10 March 2016, and #377-2017 on 13 April 2017). This secondary analysis was also approved by the UHN REBs (#23-6004 on 23 January 2024). All study procedures follow the standards of the UHN REB and the 1964 Helsinki declaration and its later amendments.

From 2016 to 2018, the primary studies only recruited adult (≥18 years) kidney transplant (KT) recipients. In 2019, recruitment was extended to adult kidney–pancreas (KP) and adult liver transplant (LT) recipients. In March 2020, recruitment was halted by the onset of the COVID-19 pandemic. In July 2021, recruitment was resumed. Exclusion criteria were severe acute illness, a diagnosis of dementia, or patients who were non-English-speaking. All participants provided informed written consent before enrollment. In this secondary analysis, we included SOTRs (KT, KP, and LT) enrolled in the primary studies, who consented for their data to be entered in the Co-PreDS database for future secondary analysis.

### 2.2. Data Sources and Measures

The participants completed questionnaires during their regular post-transplant clinic visit on tablets using an electronic data capture system (Data-Driven Outcomes System [DADOS], Techna Institute, UHN, Toronto, ON, Canada) (https://www.dadosproject.com/) [27]. If participants were off-site, a link to the study questionnaires was sent via email using DADOS Connect (Techna Institute, UHN, Toronto, ON, Canada), a secure UHN platform, which allowed participants to complete the questionnaires on their own devices.

#### 2.2.1. Sociodemographic and Clinical Characteristics

The participants were asked to self-report sociodemographic characteristics including race, age, sex, and educational level. We assessed socioeconomic status (SES) using the material deprivation domain of the Ontario Marginalization Index, which uses weighted average factor scores for each postal code in Ontario to generate quintiles from least deprived (1) to most deprived (5); we categorized quintiles into high (quintiles 1–2), middle (quintile 3), and low SES (quintiles 4–5). We collected clinical information from the patient’s medical records. Additionally, comorbid diagnoses were extracted from medical records to compute the Charlson Comorbidity Index (CCI).

Sociodemographic and clinical characteristics were ascertained at the time of or within 3 months of PROMIS questionnaire completion.

#### 2.2.2. Questionnaires

The PROMIS (HealthMeasures, Northwestern University, Chicago, IL, USA) program was funded by the United States National Institutes of Health (NIH) to develop and validate generic PROMs using item response theory (IRT), to be used by different patient populations to assess domains of physical, emotional, and social health [24].

The item banks measuring the domains of anxiety and depression used in this study are Anxiety v2.0 (29 items) and Depression v1.0 (28 items). The PROMIS-A item bank assesses fear, anxious misery, hyperarousal, and somatic symptoms related to autonomic arousal. The PROMIS-D item bank assesses negative mood, views of self, social cognition, and decreased positive affect and engagement. Patients rate the frequency with which they experienced symptoms over the past 7 days on a five-point Likert scale, with higher scores indicating a higher frequency of experiencing symptoms (from “Never” to “Always”). For each domain, T-scores range from ~10 to ~90, standardized to yield a mean (SD) of 50 (10) for the U.S. general population, with a higher T-score indicating higher symptom severity [28].

The participants completed PROMIS item banks using either PROMIS-29 or computer adaptive testing (CAT). The PROMIS-29 short form uses four items to assess anxiety and depression [29]. For PROMIS CAT, the first item targets the middle of the T-score range and is completed by all participants; subsequent items are chosen adaptively by an algorithm based on the responses to previous items until at least 4 items are completed, the standard error of measurement (SEM) is 0.30 or less (equivalent to a reliability of 0.9 or higher), or 12 items have been asked. PROMIS-29 and CAT yield nearly identical scores in SOTRs [30].

### 2.3. Exposure Assessment and Classification

The exposure of interest “Pandemic Experience” considers both the date of symptom assessment and the date of transplant relative to the onset of the COVID-19 pandemic. March 2020 was chosen as the date marking the onset of the COVID-19 pandemic, corresponding with the date the Director of the World Health Organization declared the COVID-19 outbreak a pandemic [31]. This exposure variable is coded with three categories: participants who were both transplanted and completed the PROMIS-A and PROMIS-D questionnaires prior to March 2020 (PRE), participants who were transplanted before the onset of the COVID-19 pandemic but completed the PROMIS-A and PROMIS-D assessment after pandemic onset (POST-1), and participants both transplanted and assessed after the onset of the COVID-19 pandemic (POST-2). All participants were recruited between April 2016 and April 2024. This categorization provides a pre-pandemic reference, captures the experiences of patients who were transplanted before the pandemic but are now facing pandemic-related stressors, and accounts for the additional challenges faced by patients transplanted post-pandemic onset that could potentially heighten anxiety and depression symptoms.

### 2.4. Outcome Assessment and Classification

We considered two outcomes: PROMIS-A and PROMIS-D T-scores. The PROMIS-A and PROMIS-D T-scores were derived from participants’ responses to the PROMIS-A items and PROMIS-D items, respectively, administered either as CAT or PROMIS-29.

### 2.5. Statistical Analysis

Continuous variables were summarized using mean (standard deviation [SD]) for normally distributed data and median (interquartile range [IQR]) for skewed variables (assessed by the Shapiro–Wilk test). Categorical variables were reported as counts and percentages (n, %). Continuous sociodemographic and clinical variables were compared between PRE, POST-1, and POST-2 groups using one-way ANOVA (normally distributed) or Kruskal–Wallis (skewed), and a chi-squared test was used for categorical variables (Table 1).

PROMIS-A and PROMIS-D were compared between PRE, POST-1, and POST-2 using adjusted means (95% confidence intervals—CI) derived from multivariable linear regression estimates. The dependent variables were PROMIS-A or PROMIS-D, with “Pandemic Experience” as the independent variable. Potential confounder variables (type of organ transplanted, age, sex, ethnicity, socioeconomic status, education, CCI, and time since transplantation at assessment) were included in the model based on their known associations with anxiety and/or depressive symptoms, as well as to account for differences observed across the three groups [32,33,34,35]. Post hoc pairwise comparisons were used to explore the differences between PRE, POST-1, and POST-2.

In order to better understand the clinical importance of any potential differences in anxiety or depressive symptoms, PROMIS-A and PROMIS-D T-scores were further categorized into three severity levels based on established thresholds: no symptoms (<55), mild symptoms (55–59), and moderate/severe symptoms (≥60) [36]. We present the number and proportion of participants categorized into each severity level across the three groups (PRE, POST-1, and POST-2). A multivariable ordinal logistic regression model was then applied, with PROMIS-A severity categories as the dependent variable and “Pandemic Experience” as the independent variable (reference: PRE), to estimate the odds ratios (ORs) and 95% CI for being categorized into a higher symptom severity category across groups. Potential confounder variables (type of organ transplanted, age, sex, ethnicity, socioeconomic status, education, CCI, and time since transplantation at assessment) were included in the model. The proportional odds assumption was tested and met.

We also performed a sensitivity analysis including only individuals who were assessed within one year of transplant to account for the differences in time since transplant between groups. Additionally, the interaction between “Pandemic Experience” and the type of organ transplanted was evaluated by adding an interaction term to the multivariable linear regression model. A post hoc subgroup analysis was planned if a significant interaction was identified.

Missingness was <15% for each variable and was handled using multiple imputation by chained equations, generating five imputed datasets. Outcome variables were not imputed. Estimates and standard errors were combined across datasets using Rubin’s Rule, which accounts for both within- and between-imputation variability to reflect the uncertainty introduced by missing data. All statistical analyses were executed using STATA version 15.1 (StataCorp, College Station, TX, USA).

## 3. Results

A total of 1006 participants were enrolled in the primary PROMIS validation studies, and 190 were excluded for being assessed <30 days after their transplant. A total of 816 participants were enrolled in this secondary analysis, among whom 588 (72%) were transplanted and completed an anxiety and depressive symptoms assessment before the pandemic onset (PRE), 135 (17%) were transplanted before but completed an anxiety and depressive symptoms assessment after the pandemic onset (POST-1), and 93 (11%) were transplanted and completed an anxiety and depressive symptoms assessment after the pandemic onset (POST-2) (Table 1). PROMIS-29 was completed by 132 patients, who were all kidney transplant patients and in the PRE group (22%). The participants in the POST-1 group were moderately older. The distribution of sex and SES was similar between the three groups. The distribution of type of transplanted organ was different between the PRE, POST-1, and POST-2 groups: in the PRE group, 404 (69%) were KT, 36 (6%) were KP, and 148 (25%) were LT, compared with 42 (31%) KT, 58 (43%) KP, and 35 (26%) LT in the POST-1 group, and 41 (44%) KT, 25 (27%) KP, and 27 (29%) LT in the POST-1 group (*p* < 0.001). This higher proportion of KP in POST-1 and POST-2 may be the cause of the higher proportion of comorbidities in POST-1 and POST-2 compared with PRE (Table 1). There were no significant differences between patients who completed PROMIS-29 or PROMIS CAT, and domain scores were similar across both sources.

Unadjusted PROMIS-A and PROMIS-D scores across the three groups are presented in Table S1. In the fully adjusted model that included the type of organ transplanted, age, sex, ethnicity, socioeconomic status, education, CCI, and time since transplantation at assessment, the adjusted mean (95% CI) PROMIS-A T-scores were 51.7 (50.9; 52.4) in PRE, 52.6 (51.0; 54.3) in POST-1, and 54.2 (52.3; 54.3) in POST-2 (Table 2). The pairwise comparisons of the PROMIS-A scores indicated that the mean PROMIS A score was not significantly different between PRE vs. POST-1, and POST-1 vs. POST-2. However, the POST-2 group had significantly higher PROMIS-A scores compared with PRE (adjusted mean [95% CI]: 54.2 [52.3; 56.1] vs. 51.7 [50.9; 52.4], *p* = 0.02). A post hoc power analysis estimated 69% power to detect the observed difference between POST-2 and PRE. No significant interaction was found between the type of organ transplanted and “Pandemic Experience” (*p*-value for interaction = 0.7); therefore, we did not conduct subgroup analysis by organ type. In a sensitivity analysis limited to individuals assessed within one year of transplant (as a result, only the PRE and POST-2 groups were eligible) (Table S2), the POST-2 group still had significantly higher PROMIS-A scores compared with the PRE group (adjusted mean [95% CI]: 55.5 [53.3; 57.7] vs. 51.3 [49.7; 52.9], *p* = 0.003).

The distribution of anxiety symptom severity categories across the Pandemic Experience groups is shown in Table 3. The number (%) of SOTRs categorized as having no symptoms were 367 (62%), 85 (63%), and 46 (49%) in the PRE, POST-1, and POST-2 groups, respectively. Those categorized as having mild symptoms were 116 (20%), 31 (23%), and 22 (24%) in the PRE, POST-1, and POST-2 groups, respectively. For moderate/severe symptoms, the proportions were 105 (18%), 19 (14%), and 25 (27%) in the PRE, POST-1, and POST-2 groups, respectively. The multivariable ordinal logistic regression analysis revealed that, compared with the PRE group, participants in the POST-2 group were more likely to be categorized as having more severe anxiety symptoms (OR: 1.59, 95% CI: 1.0; 2.5, *p* = 0.048), a finding that was borderline statistically significant. This association remained significant in the sensitivity analysis limited to individuals assessed within one year of transplant (OR: 2.6, 95% CI: 1.3; 5.2, *p* = 0.005) (Table S3). The odds for the POST-1 group were not different compared with the PRE group.

In the fully adjusted model, the adjusted mean (95% CI) PROMIS-D scores were 49.6 (48.8; 50.3) in PRE, 49.7 (48.0; 51.3) in POST-1, and 49.9 (48.1; 51.8) in POST-2 (Table 2). The pairwise comparisons indicated that the mean PROMIS-D scores were not significantly different in these groups. Additionally, no significant interaction was found between the type of organ transplanted and “Pandemic Experience” (*p*-value for interaction = 0.4). The proportions categorized as having no symptoms were 437 (74%), 98 (73%), and 58 (62%) in the PRE, POST-1, and POST-2 groups, respectively. Those categorized as having mild symptoms were 72 (12%), 17 (13%), and 17 (18%) in the PRE, POST-1, and POST-2 groups, respectively. For moderate symptoms, the proportions were 75 (13%), 18 (13%), and 16 (17%) in the PRE, POST-1, and POST-2 groups, respectively. Finally, those categorized as having severe symptoms were 4 (1%), 2 (1%), and 2 (2%) in the PRE, POST-1, and POST-2 groups, respectively. The multivariable ordinal logistic regression analysis showed no significant differences in depression symptom severity categories between the PRE, POST-1, and POST-2 groups.

## 4. Discussion

In this study using a repeated cross-sectional design, we assessed whether SOTRs had worse anxiety and depressive symptoms after the COVID-19 pandemic onset. We found more severe anxiety but not depression symptoms post-pandemic onset. Specifically, we found that participants who were both transplanted and completed symptom assessment after the pandemic onset had significantly higher PROMIS-A scores and were more likely to have more severe anxiety symptoms compared with participants who were transplanted and completed symptom assessment before the pandemic onset. The PROMIS-D scores were not different across the exposure groups.

The COVID-19 pandemic has been shown to be associated with worse mental health across various populations, with increased anxiety and depression symptoms reported among both general and high-risk groups, including individuals with type 2 diabetes, patients undergoing cardiac rehabilitation, and those with chronic illnesses such as systemic sclerosis [37,38,39,40,41,42,43,44]. In our sample of SOTRs, we consider the difference in PROMIS-A scores observed between the Pandemic Experience groups to be clinically potentially meaningful, since the reported minimally clinically important difference for PROMIS domain scores ranges 2–5 points [45,46,47,48,49,50,51]. Furthermore, there seemed to be higher proportions of participants categorized as having mild or moderate/severe anxiety symptoms in the POST-2 group compared with the pre-pandemic reference group (Table 3). This difference appeared to be more pronounced among those who were assessed within one year since transplant (Table S3). Our findings align with published reports about anxiety and depression symptoms among SOTRs following the pandemic onset [16,17]. Specifically, a longitudinal study of anxiety and depressive symptoms among KT recipients found worsened anxiety symptoms but improved depression symptoms over time during a two-year period after pandemic onset [16].

One potential explanation for this difference between the pattern of anxiety versus depression may be attributed to the nature of the pandemic-induced stressors and how they are processed. The abrupt and pervasive threats posed by the virus likely triggered a more reactive form of stress among SOTRs, leading to heightened anxiety, as reflected in the increased PROMIS-A scores observed in our study. PROMIS-A primarily evaluates symptoms related to autonomic arousal and threat perception, which are often driven by the acute activation of biological stress systems such as the hypothalamic–pituitary–adrenal axis [52]. Psychosocial stressors like health risks and the perceived scarcity of educational resources, information, and guidance specific to COVID-19 for immunocompromised individuals could have rapidly triggered these systems, leading to amplified anxiety among this population [18].

On the other hand, the more chronic aspects of depression measured by PROMIS-D item banks (negative mood, self-criticism, social cognition, and decreased positive affect and engagement) [53] might not have been as significantly affected by the pandemic’s acute stressors among SOTRs, and may be more influenced by sustained disruptions or a lack of meaning, often unfolding over time. It is also possible that despite the limitations in healthcare access, many patients maintained access to social and emotional support at home. This support system, through family members or caregivers, may have played a protective role against depressive symptoms, but did not address the acute stress and uncertainty driving anxiety.

In fact, in the ‘Improving Engagement and Empowering Patients on Their Transplant Journey’ report by the Kidney Foundation of Canada [54], a prominent theme voiced by patients throughout their transplant journey was “navigating uncertainty” and “never knowing when the next challenge would arise”. Our observed patterns of higher anxiety symptoms—rather than depression—may reflect this theme, particularly in the context of pandemic-related infection risk and disrupted care.

While anxiety is often elevated around the time of transplant surgery—and the POST-2 group had a shorter time between transplant and mental health assessment compared with other groups—all participants in our study were assessed at least one month post transplant. The findings from our prior longitudinal work among recently transplanted patients support that anxiety symptoms typically return to normal levels within 4–6 weeks after transplant [55]. Also, although not statistically significant, the POST-2 group had elevated OR for moderate/severe depression symptoms, a trend that may merit further investigation.

Our three-level “Pandemic Experience” exposure (PRE, POST-1, and POST-2) method offers a comprehensive framework to examine anxiety and depressive symptoms among SOTRs during the pandemic. PRE serves as a pre-pandemic reference, while POST-1 and POST-2 capture the relevant experiences of transplant recipients navigating pandemic-related stressors. Specifically, POST-1 reflects established SOTRs transplanted prior to the pandemic who are now navigating pandemic-related stressors, while POST-2 represents patients who were transplanted amidst these stressors, which could significantly impact anxiety and depression symptoms. We saw that anxiety symptoms were progressively worse from PRE to POST-1 to POST-2. This difference in anxiety levels can be explained by the degree of exposure to pandemic-related stresses and the perceived threat presented by a potential COVID-19 infection.

The participants in the POST-1 group were experiencing the impact of the pandemic as stable transplant recipients, already familiar with the anti-rejection medications, potential side effects, and their treatments at the time of assessment. In contrast, POST-2 participants faced the additional impact of COVID-19 related stresses in the context of a major life event—a recent transplant—and faced added concerns due to the pandemic, with the added concerns of new medications and concerns/fears of infections, in general, and COVID-19, in particular. The POST-1 participants had a chance to adapt to their immunocompromised condition and encountered the threat of the virus with established coping mechanisms, while the POST-2 group had to adapt to both their new immunocompromised condition and the threats posed by the spreading virus. The pandemic also directly affected the transplantation and recovery experience. For instance, transplant care in Canada was significantly impacted during the pandemic [56]. Patients reported difficulties associated with hospital stays without the presence of family and support systems, due to hospital restrictions, which led to feelings of isolation [18]. Diminished support in such a critical period can moderate the extent of anxiety symptoms among transplant recipients [57], and may be one potential reason why the participants in the POST-2 group had worse anxiety symptoms compared with the participants in the POST-1 group. Other potential contributing factors include limited access to the healthcare team during both transplant workup and the continuity of care after the pandemic onset, which could have led to increased fear and reduced reassurance for the patients [57].

This study has several strengths. First, we assessed a clinically and ethnically diverse sample of SOTRs participants. Additionally, our large sample size enabled adjustment for several potential confounders. We also had a large pre-pandemic cohort as a reference. While this reference sample had relatively more KT and fewer KP recipients compared with the post-pandemic onset cohort, we adjusted for organ type in the multivariable regression analyses. Additionally, we included an interaction term for organ type in the model, which was found to be insignificant.

However, the limitations of our study need to be considered when interpreting the results. This was a convenience cohort of clinically stable patients assessed > 30 days after transplant, thus excluding those with more complex or unstable medical conditions. Moreover, the repeated cross-sectional design does not allow for the analysis of changes within the same participants over time. Additionally, our exclusion of non-English-speaking patients may limit the generalizability of our results. Given that PROMIS tools are available in over 30 languages, future research should aim to include linguistically diverse samples. We defined the categories based on the recommended PROMIS thresholds to support clinical interpretability, but this approach may have limited statistical precision and should be interpreted alongside the continuous score comparisons presented in our models. Furthermore, the POST-2 group had a relatively smaller sample size compared with other groups, potentially limiting the power to detect meaningful differences. Finally, our sample did not include all types of SOTR patients, such as heart or lung transplants, which may restrict the direct generalizability of our findings to those populations. While we adjusted for the organ type and comorbidity burden in our models, the imbalance in these variables across exposure groups raises the possibility of residual confounding that could not be fully accounted for.

In conclusion, our study found that SOTRs transplanted after the pandemic onset had worse anxiety symptoms but similar depression symptoms compared with prior to the pandemic. These findings add to the limited literature on the mental health of immunocompromised individuals during periods of heightened stress and infectious risk. While the exact circumstances of the COVID-19 pandemic may not recur, future global health threats, whether pandemics or other widespread disruptions, are possible. Our results underscore the emotional vulnerability of this population during such events, and support the importance of routine mental health screening, particularly for newly transplanted patients during periods of elevated societal stress. Integrating PROMs into post-transplant care may facilitate the timely identification and management of psychological challenges in a more structured and responsive manner.

## Figures and Tables

**Table 1:** Patient characteristics.

Characteristic	PRE (n = 588)	POST-1 (n = 135)	POST-2 (n = 93)	*p*-Value
Age (years); mean (SD)	52 (16)	58 (12)	51 (13)	<0.001
Sex (male); n (%)	369 (63)	86 (64)	54 (58)	0.6
Organ Replacement Type n (%)				<0.001
Kidney	404 (69)	42 (31)	41 (44)	
Kidney–Pancreas	36 (6)	58 (43)	25 (27)	
Liver	148 (25)	35 (26)	27 (29)	
Education (<12 yrs); n (%)	191 (34)	22 (20)	23 (30)	0.02
Socioeconomic Status n (%)				0.5
Low	232 (39)	41 (36)	29 (41)	
Middle	139 (24)	22 (19)	14 (20)	
High	216 (37)	51 (45)	28 (39)	
Albumin, g/L mean (SD)	42 (3)	42 (5)	41 (6)	0.2
CCI ≥ 4, n (%)	199 (36)	81 (65)	48 (57)	<0.001
Time since transplant, yrs; median (IQR)	6.8 (1.5; 11.8)	9.16 (4.9; 17.3)	0.5 (0.1; 1.3)	<0.001
Racialized status n (%)				0.01
White	353 (62)	91 (78)	50 (66)	
Asian	115 (20)	21 (18)	17 (22)	
Black	51 (9)	1 (1)	5 (7)	
Other	47 (8)	3 (3)	4 (5)	

SD: standard deviation; IQR: interquartile range; CCI: Charlson Comorbidity Index. PRE (transplanted and mental health assessed before COVID-19 pandemic onset), POST-1 (transplanted before pandemic onset, mental health assessed after pandemic onset), and POST-2 (transplanted and mental health assessed after pandemic onset). SES: Socioeconomic status determined using the material deprivation domain of the Ontario Marginalization Index, which uses weighted average factor scores for each postal code in Ontario to generate quintiles from least deprived (1) to most deprived (5), and we categorized quintiles into high (quintiles 1–2), middle (quintile 3), and low (quintiles 4–5) SES.

**Table 2:** The association of PROMIS-A and PROMIS-D T-scores with Pandemic Experience, evaluated using multivariable linear regression—final model, adjusted for organ type, age, sex, ethnicity, education, SES, CCI, and time since transplant.

	N	Adjusted Mean (95% CI)	*p*-Value
			Pairwise Comparisons
PROMIS-A			
PRE	588	51.7 (50.9; 52.4)	PRE vs. POST-1: *p* = 0.4
POST-1	135	52.6 (51.0; 54.3)	POST-1 vs. POST-2: *p* = 0.2
POST-2	93	54.2 (52.3; 56.1)	PRE vs. POST-2: *p* = 0.02
PROMIS-D			
PRE	588	49.6 (48.8; 50.3)	PRE vs. POST-1: *p* = 0.9
POST-1	135	49.7 (48.0; 51.3)	POST-1 vs. POST-2: *p* = 0.7
POST-2	93	49.9 (48.1; 51.8)	PRE vs. POST-2: *p* = 0.8

PRE (transplanted and mental health assessed before COVID-19 pandemic onset), POST-1 (transplanted before pandemic onset, mental health assessed after pandemic onset), and POST-2 (transplanted and mental health assessed after pandemic onset). PROMIS-A—PROMIS Anxiety; PROMIS-D—PROMIS Depression; CCI—Charlson Comorbidity Index; CI—Confidence Interval; SES—Socioeconomic status determined using the material deprivation domain of the Ontario Marginalization Index, which uses weighted average factor scores for each postal code in Ontario to generate quintiles from least deprived (1) to most deprived (5); we categorized quintiles into high (quintiles 1–2), middle (quintile 3), and low SES (quintiles 4–5).

**Table 3:** The association of PROMIS-A and PROMIS-D clinical symptom severity categories with Pandemic Experience, evaluated using multivariable ordinal logistic regression—adjusted for organ type, age, sex, ethnicity, education, SES, CCI, and time since transplant.

	No Symptoms n (%)	Mild Symptoms n (%)	Moderate/ Severe Symptoms n (%)	OR (95% CI)	*p*-Value
PROMIS-A					
PRE	367 (62)	116 (20)	105 (18)	Ref	Ref
POST-1	85 (63)	31 (23)	19 (14)	1.02 (0.7; 1.6)	0.9
POST-2	46 (49)	22 (24)	25 (27)	1.59 (1.0; 2.5)	0.048
PROMIS-D					
PRE	437 (74)	72 (12)	79 (14)	Ref	Ref
POST-1	98 (73)	17 (13)	20 (14)	1.1 (0.7; 1.8)	0.7
POST-2	58 (62)	17 (18)	18 (19)	1.49 (0.92; 2.4)	0.1

PRE (transplanted and mental health assessed before COVID-19 pandemic onset), POST-1 (transplanted before pandemic onset, mental health assessed after pandemic onset), and POST-2 (transplanted and mental health assessed after pandemic onset). PROMIS-A—PROMIS Anxiety; PROMIS-D—PROMIS Depression; CCI—Charlson Comorbidity Index; CI—Confidence Interval; SES—Socioeconomic status determined using the material deprivation domain of the Ontario Marginalization Index, which uses weighted average factor scores for each postal code in Ontario to generate quintiles from least deprived (1) to most deprived (5); we categorized quintiles into high (quintiles 1–2), middle (quintile 3), and low SES (quintiles 4–5). OR—Odds Ratio from the multivariable ordinal logistic regression represents the odds of being in a higher symptom severity category for each level of Pandemic Exposure compared with reference (PRE). Symptom severity categories are defined as follows: No Symptoms (T-score < 55), Mild Symptoms (T-score 55–59), and Moderate/Severe Symptoms (T-score ≥ 60).

## Data Availability

The data supporting this study are not publicly available due to privacy and confidentiality restrictions. However, de-identified data may be made available upon reasonable request to the corresponding author, subject to institutional and ethical approvals.

## Supplementary Materials

The following supporting information can be downloaded at: https://www.mdpi.com/article/10.3390/jcm14144920/s1, Table S1: The association of PROMIS-A and PROMIS-D T-scores with Pandemic Experience, evaluated using multivariable linear regression—unadjusted model; Table S2: Sensitivity analysis for the association between PROMIS-A T-scores and Pandemic Experience in individuals assessed within 1 year of transplant, evaluated using multivariable linear regression—model adjusted for organ type, age, sex, ethnicity, education, SES, CCI, and time since transplant; Table S3: Sensitivity for the association of PROMIS-A clinical symptom severity categories with Pandemic Experience in individuals assessed within 1 year of transplant, evaluated using multivariable ordinal logistic regression—model adjusted for organ type, age, sex, ethnicity, education, SES, CCI, and time since transplant; Figure S1: Patient flow diagram.

## Abbreviations

The following abbreviations are used in this manuscript:

ANOVA	Analysis of Variance
CAT	Computer Adaptive Testing
CCI	Charlson Comorbidity Index
CI	Confidence Interval
Co-PreDS	Comprehensive Psychosocial Research Data System
DADOS	Data-Driven Outcomes System
IQR	Interquartile Range
IRT	Item Response Theory
KHERG	Kidney Health Education and Research Group
KP	Kidney–Pancreas
KT	Kidney Transplant
LT	Liver Transplant
NIH	National Institutes of Health
OMI	Ontario Marginalization Index
POST	Assessment period after pandemic onset
POST-1	Transplanted before and assessed after pandemic onset
POST-2	Transplanted and assessed after pandemic onset
PRE	Assessment period before pandemic onset
PROM	Patient-Reported Outcome Measure
PROMIS	Patient-Reported Outcomes Measurement Information System
PROMIS-A	PROMIS Anxiety
PROMIS-D	PROMIS Depression
REB	Research Ethics Board
SD	Standard Deviation
SEM	Standard Error of Measurement
SES	Socioeconomic Status
SOTRs	Solid Organ Transplant Recipients
UHN	University Health Network
