# Supplementary material for: Anxiety and Depressive Symptoms Post-COVID-19 Pandemic Onset in Solid Organ Transplant Recipients: Canadian Repeated Cross-Sectional Study
Source: J Clin Med. 2025 Jul 11;14(14):4920. doi: 10.3390/jcm14144920 (PMC12295447; doi:10.3390/jcm14144920)
Supplement: Supplementary file 1 [file jcm-14-04920-s001.zip › jcm-3681297-supplementary.pdf]

Table S1: The association of PROMIS-A and PROMIS-D T-scores with Pandemic Experience, evaluated using multivariable linear regression - unadjusted model

Domain	Group	N	Adjusted Mean (95% CI)	P- value<br>Pairwise Comparisons
**PROMIS-A**	PRE	588	51.7 (50.9; 52.4)	PRE vs POST-1: p=0.4
	POST-1	135	52.3 (50.8; 53.8)	POST-1 vs POST-2: p=0.08
	POST-2	93	54.4 (52.6; 56.3)	PRE vs POST-2: p=0.006
**PROMIS-D**	PRE	588	49.3 (48.5; 50.0)	PRE vs POST-1: p=0.3
	POST-1	135	50.1 (48.6; 51.6)	POST-1 vs POST-2: p=0.7
	POST-2	93	50.6 (48.8; 52.4)	PRE vs POST-2: p=0.3

PRE (transplanted and mental health assessed before COVID-19 pandemic onset), POST-1 (transplanted before, mental health assessed after pandemic onset), POST-2 (transplanted and mental health assessed after pandemic onset).
PROMIS-A: PROMIS Anxiety; PROMIS-D: PROMIS Depression; CCI: Charlson Comorbidity Index, SES: Socioeconomic status determined using the material deprivation domain of the Ontario Marginalization Index, which uses weighted average factor scores for each postal code in Ontario to generate quintiles from least deprived (1) to most deprived (5), and we categorized quintiles into high (quintiles 1-2), middle (quintile 3) and low SES (quintiles 4-5).

Table S2: Sensitivity analysis for the association between PROMIS-A T-scores and Pandemic Experience in individuals assessed within 1 year of transplant, evaluated using multivariable linear regression – model adjusted for organ type, age, sex, ethnicity, education, SES, CCI, time since transplant

Domain	Group	N	Adjusted Mean (95% CI)	P- value
**PROMIS-A**	PRE	119	51.3 (49.7; 52.9)	Ref
	POST-1	0	NA	NA
	POST-2	58	55.5 (53.3; 57.7)	0.003

PRE (transplanted and mental health assessed before COVID-19 pandemic onset), POST-1 (transplanted before, mental health assessed after pandemic onset), POST-2 (transplanted and mental health assessed after pandemic onset).

PROMIS-A: PROMIS Anxiety; CCI: Charlson Comorbidity Index, SES: Socioeconomic status determined using the material deprivation domain of the Ontario Marginalization Index, which uses weighted average factor scores for each postal code in Ontario to generate quintiles from least deprived (1) to most deprived (5), and we categorized quintiles into high (quintiles 1-2), middle (quintile 3) and low SES (quintiles 4-5).

Table S3: Sensitivity for the association of PROMIS-A clinical symptom severity categories with Pandemic Experience in individuals assessed within 1 year of transplant, evaluated using multivariable ordinal logistic regression – model adjusted for organ type, age, sex, ethnicity, education, SES, CCI, time since transplant

	**No Symptoms** n (%)	**Mild Symptoms** n (%)	**Moderate/ Severe Symptoms** n (%)	OR (95% CI)	P-value
**PROMIS-A**					
PRE	74 (62)	28 (24)	17 (14)	Ref	Ref
POST-1	0 (0)	0 (0)	0 (0)	NA	NA
POST-2	26 (46)	16 (27)	16 (27)	2.7 (1.4; 5.5)	0.003

PRE (transplanted and mental health assessed before COVID-19 pandemic onset), POST-1 (transplanted before, mental health assessed after pandemic onset), POST-2 (transplanted and mental health assessed after pandemic onset).

PROMIS-A: PROMIS Anxiety; CCI: Charlson Comorbidity Index, SES: Socioeconomic status determined using the material deprivation domain of the Ontario Marginalization Index, which uses weighted average factor scores for each postal code in Ontario to generate quintiles from least deprived (1) to most deprived (5), and we categorized quintiles into high (quintiles 1-2), middle (quintile 3) and low SES (quintiles 4-5).

OR: Odds Ratio from the multivariable ordinal logistic regression represents the odds of being in a higher symptom severity category for each level of Pandemic Exposure compared to reference (PRE); CI: Confidence Interval

Symptom severity categories are defined as follows: No Symptoms (T-score < 55), Mild Symptoms (T-score 55–59), Moderate Symptoms (T-score 60–69), and Severe Symptoms (T-score ≥ 70).

Supplementary Material

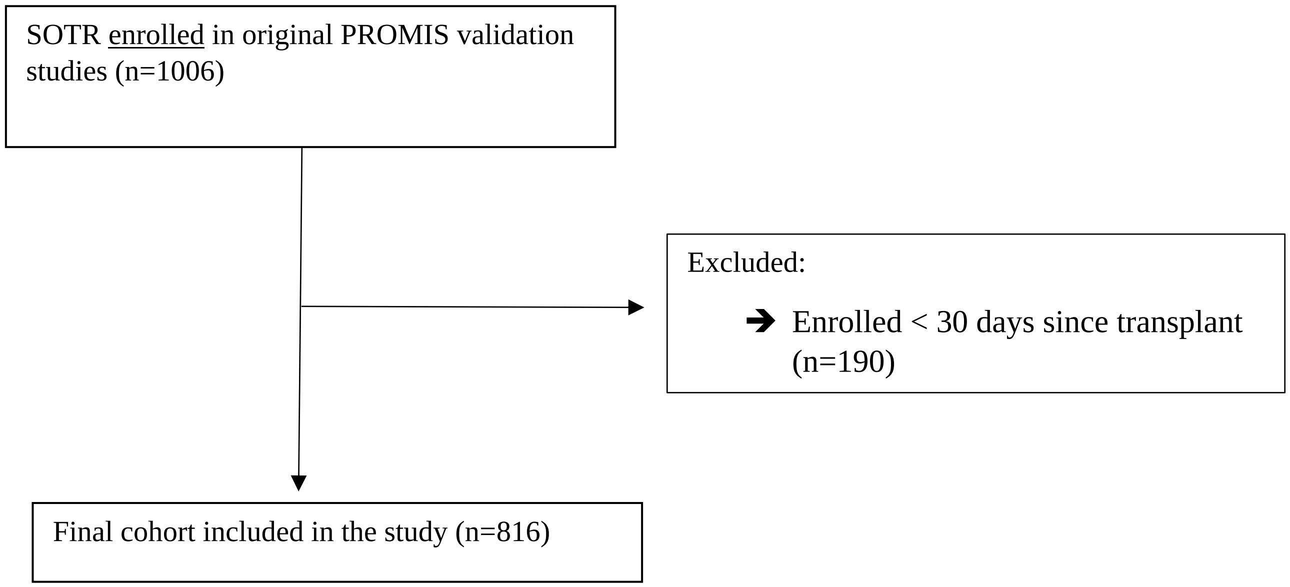

Figure S1: Patient flow diagram
SOTR: Solid organ transplant recipients; PROMIS: Patient Reported Outcomes Measurement Information System
